# Hypoxia-inducible factor prolyl hydroxylase 1 (PHD1) deficiency promotes hepatic steatosis and liver-specific insulin resistance in mice

**DOI:** 10.1038/srep24618

**Published:** 2016-04-20

**Authors:** Amandine Thomas, Elise Belaidi, Judith Aron-Wisnewsky, Gerard C. van der Zon, Patrick Levy, Karine Clement, Jean-Louis Pepin, Diane Godin-Ribuot, Bruno Guigas

**Affiliations:** 1 Laboratoire HP2, Université Grenoble Alpes, Grenoble, F-38042 France; 2 INSERM U1042, Grenoble, F-38042 France; 3 Institute of Cardiometabolism and Nutrition (ICAN), Assistance Publique Hôpitaux de Paris, Pitié-Salpêtrière Hospital, Paris, France; 4 INSERM, U1166, Nutriomics Team 6, Paris, France; 5 Sorbonne Universités, UPMC University Paris 06, UMR_S 1166, Nutriomics Team, Paris, France; 6 Department of Molecular Cell Biology, Leiden University Medical Center, Leiden, The Netherlands; 7 Department of Parasitology, Leiden University Medical Center, Leiden, The Netherlands

## Abstract

Obesity is associated with local tissue hypoxia and elevated hypoxia-inducible factor 1 alpha (HIF-1α) in metabolic tissues. Prolyl hydroxylases (PHDs) play an important role in regulating HIF-α isoform stability. In the present study, we investigated the consequence of whole-body PHD1 gene (*Egln2*) inactivation on metabolic homeostasis in mice. At baseline, PHD1−/− mice exhibited higher white adipose tissue (WAT) mass, despite lower body weight, and impaired insulin sensitivity and glucose tolerance when compared to age-matched wild-type (WT) mice. When fed a synthetic low-fat diet, PHD1−/− mice also exhibit a higher body weight gain and WAT mass along with glucose intolerance and systemic insulin resistance compared to WT mice. PHD1 deficiency led to increase in glycolytic gene expression, lipogenic proteins ACC and FAS, hepatic steatosis and liver-specific insulin resistance. Furthermore, gene markers of inflammation were also increased in the liver, but not in WAT or skeletal muscle, of PHD1−/− mice. As expected, high-fat diet (HFD) promoted obesity, hepatic steatosis, tissue-specific inflammation and systemic insulin resistance in WT mice but these diet-induced metabolic alterations were not exacerbated in PHD1−/− mice. In conclusion, PHD1 deficiency promotes hepatic steatosis and liver-specific insulin resistance but does not worsen the deleterious effects of HFD on metabolic homeostasis.

Obesity is believed to be associated with some degree of hypoxia in metabolic organs, notably in white adipose tissue (WAT)^1^,^2^. A significant decrease in oxygen tension (pO_2_) was indeed reported in various fat pads from different mouse models of obesity and in subcutaneous WAT from overweight and obese subjects^3^, although this later observation still remains a matter of discussion^4^. Hypoxia is suggested to be involved in WAT inflammation^5^ and to play a major role in obesity-induced metabolic dysfunctions, at least partly by impairing adipocyte insulin sensitivity^6^,^7^. At the mechanistic level, WAT hypoxia promotes the increase in both gene and protein expression of hypoxia-inducible transcription factors (HIFs)^8^. HIFs are heterodimers composed of the constitutive HIF-β nuclear subunit and of one of three isoforms of the O_2_-regulated HIF-α cytosolic subunit, namely HIF-1α, HIF-2α and HIF-3α^9^,^10^. HIF-α expression and transcriptional activity are tightly regulated by both the oxygen-sensitive factor inhibiting HIF (FIH) and members of the prolyl hydroxylase (PHD) family^9^,^10^. PHDs are evolutionary conserved dioxygenases that require oxygen and 2-oxoglutarate as co-substrates and iron and ascorbic acid as cofactors for hydroxylation of specific proline residues on HIF-α. Three PHD isoforms have been identified in mammals (PHD1, PHD2, and PHD3) and are ubiquitously expressed in most tissues^9^,^10^, although their respective roles in the control of tissue-specific levels of HIF-α isoforms remain to be clarified^11^. In normoxic conditions, PHDs act as oxygen-sensing enzymes and promote hydroxylation of HIF-α subunits, allowing recognition by the protein von Hippel-Lindau (pVhl) ubiquitin ligase complex and subsequent proteasomal degradation^9^,^10^. In contrast, PHD activity is reduced during hypoxia, leading to stabilization of HIF-α subunits and their subsequent translocation to the nucleus where they can dimerize with HIF-β, subsequently triggering the transcription of specific target genes^12^. Among the various HIFs, the HIF-1 transcription factor appears to play a major role in obesity-associated insulin resistance and metabolic dysfunctions, notably by promoting expression and secretion of chemokines/adipokines and recruitment of pro-inflammatory macrophages and T cell accumulation in hypoxic WAT^2^. In contrast, pharmacological or genetic inhibition of HIF-1α was reported to decrease high-fat diet-induced metabolic dysfunctions^13^,^14^,^15^,^16^,^17^, as shown by attenuated adipose tissue fibrosis and inflammation^13^ and reduced hepatic steatosis^14^. Overall, this indicates that modulation of HIF-1 transcriptional activity in metabolic tissues might be involved in the hypoxia- and obesity-associated metabolic alterations. Various genetically engineered mouse models for whole-body and tissue-specific inactivation of the three PHD isoforms have been developed^18^,^19^,^20^ but the exact impact of such deficiencies on whole-body metabolic homeostasis remains unclear^1^ and no data are currently available in PHD1-deficient mice.

In the present study, we aimed to investigate the consequences of constitutive inactivation of the PHD1 gene (*Egln2*), one of the critical component of the HIF-PHD oxygen-sensing pathway, on adiposity, whole-body glucose homeostasis and tissue specific insulin-sensitivity on both low (LFD) and high-fat (HFD) diet.

## Results

### PHD1 deficiency impairs whole-body metabolic homeostasis

We first determined the effect of constitutive deletion of the PHD1 gene on expression of the other PHD isoforms and of the HIF-1 target gene *Ldha* in liver, epididymal white adipose tissue (eWAT) and skeletal muscle (Fig. S1). As expected, the expression of the full length *Egln2* mRNA coding for the active PHD1 protein was undetectable in each tissue. In the liver, an increase in the expression of *Egln3* (PHD3) was observed (Fig. S1A), while an upregulation of *Egln1* (PHD2) and a downregulation of *Egln3* were evidenced in eWAT (Fig. S1B). No effect of *Egln2* (PHD1) deletion on the expression of other PHDs isoforms was detected in skeletal muscle (Fig. S1C), in line with a previous report^21^. The expression of *Ldha*, a HIF target gene, was elevated in liver and WAT from PHD1-deficient mice, suggesting increased HIFs transcriptional activities in these tissues (Fig. S1A,B).

**Figure 1:**
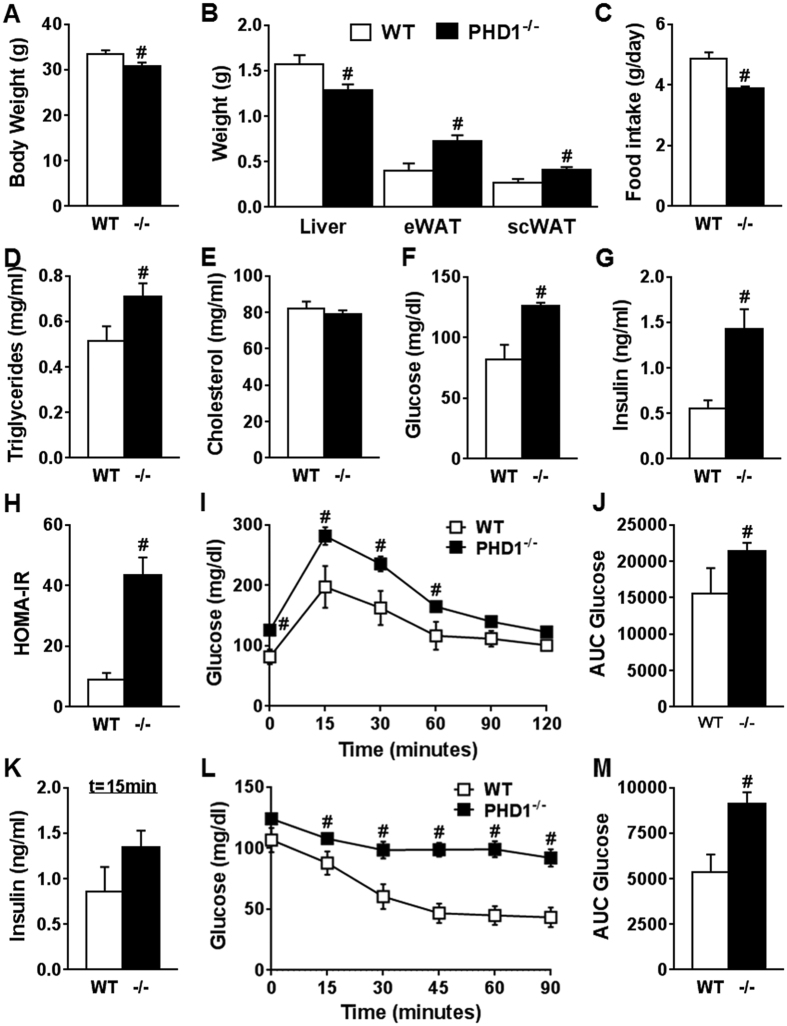
PHD1 deficiency induces glucose intolerance and insulin resistance in standard chow-fed mice. Body weight (**A**) and weights of liver, epididymal white adipose tissue (eWAT) and subcutaneous white adipose tissue (scWAT) were measured at sacrifice in 12 week-old WT (open bars) and PHD1−/− (black bars) mice on standard chow diet (**B**). Daily food intake (**C**) was monitored during 5 weeks. Fasting plasma triglycerides (**D**), total cholesterol (**E**), glucose (**F**) and insulin (**G**) levels were determined in 6-hour unfed mice and HOMA-IR (**H**) was calculated. An intraperitoneal glucose tolerance test (ipGTT, 2 g/kg total body weight) was performed in 6-hour unfed mice. Blood glucose levels were measured at the indicated time-points (**I**), and the AUC of the glucose excursion curve was calculated as a surrogate for whole-body glucose tolerance (**J**). The plasma insulin level during ipGTT was measured at 15 minutes (**K**). An intraperitoneal insulin tolerance test (0.5 U/kg total body weight) was performed in 4-hour unfed mice. Blood glucose levels were measured at the indicated time-points (**L**), and the AUC of the glucose excursion curve was calculated as a surrogate for whole-body insulin sensitivity (**M**). Data are means ± SEM (n = 7 for WT; n = 13 for PHD1−/−). ^#^p < 0.05 *vs* WT mice.

To investigate the role of PHD1 on whole-body metabolic homeostasis, 12 week-old PHD1-deficient (PHD1−/−) and WT control mice on regular chow diet were first compared. PHD1−/− mice exhibited lower body and liver weights (−8 and −18%, respectively; p < 0.05; Fig. 1A,B) and higher epididymal and subcutaneous adipose tissue weights (+83 and +58%, respectively; p < 0.05; Fig. 1A,B) compared to WT mice, suggesting that PHD1 controls adiposity. In link with decreased body weight, mean daily food intake was also lower in PHD1−/− compared to WT mice (Fig. 1C). Despite reduced body weight, PHD1 deficiency was associated with elevated fasting plasma triglycerides (TG) (Fig. 1D), glucose (Fig. 1F) and insulin (Fig. 1G) levels, whereas total cholesterol (TC) was unchanged (Fig. 1E). The calculated HOmeostasis Model Assessment of Insulin Resistance index (HOMA-IR) adjusted for rodents was increased in PHD1−/− mice compared to WT mice (Fig. 1H), suggesting systemic insulin resistance. To assess the effect of PHD1 deletion on whole-body glucose homeostasis and insulin sensitivity, mice were next subjected to intraperitoneal glucose (GTT) and insulin tolerance tests (ITT), respectively. In line with increased HOMA-IR, PHD1−/− mice displayed impaired whole-body glucose tolerance (Fig. 1I–K) and insulin sensitivity (Fig. 1L,M) compared to WT mice.

### PHD1 deficiency promotes body weight and fat mass gain, dyslipidemia and glucose intolerance but does not worsen HFD-induced metabolic alterations

**Figure 2:**
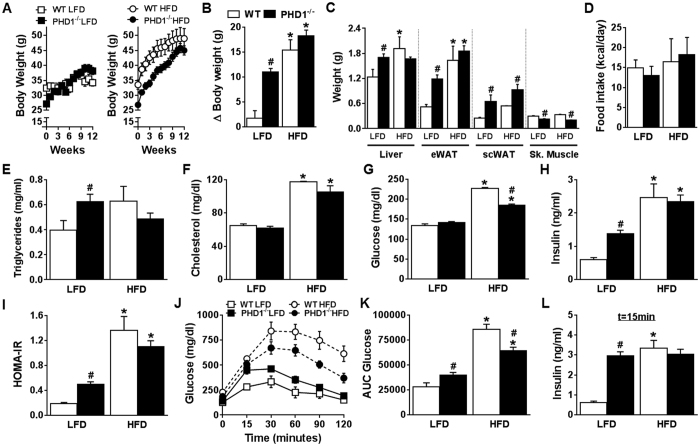
PHD1 deficiency promotes weight gain and insulin resistance but does not worsen high fat diet-induced metabolic alterations. WT (open bars) and PHD1−/− (black bars) mice were fed a low-fat (LFD, 10% fat) or high-fat (HFD, 45% fat) diet for 12 weeks. Body weight was monitored throughout the experimental period (**A**). Delta (Δ) change in body weight from the start of diet (**B**), weight of liver, epididymal (eWAT) and subcutaneous (scWAT) white adipose tissue, and skeletal (Sk.) muscle (**C**) were measured after sacrifice at week 12. Mean daily food intake (**D**) was recorded during 12 weeks. At week 12, plasma triglycerides (**E**), total cholesterol (**F**), glucose (**G**) and insulin levels (**H**) were measured in 6-hour unfed mice and HOMA-IR (**I**) was calculated. An intraperitoneal GTT (2 g/kg of total body weight) was performed in 6-hour unfed mice at week 11. Blood glucose levels were measured at the indicated time-points (**J**), and the area under the curve (AUC) of the glucose excursion curve was calculated as a measure of glucose tolerance (**K**). The plasma insulin level during ipGTT was measured at 15 minutes (**L**). Data are means ± SEM (n = 4 for LFD-WT; n = 7 for LFD-PHD1−/−; n = 5 for HFD-WT; n = 7 for HFD-PHD1−/−). *p < 0.05 *vs* LFD-fed mice, ^#^p < 0.05 *vs* WT mice.

To further investigate the impact of PHD1 deletion on metabolic homeostasis, WT and PHD1−/− mice were next challenged with synthetic low- (LFD, 10% kcal from fat) or high-fat (HFD, 60% kcal from fat) diets for 12 weeks. PHD1−/− mice fed a LFD for 12 weeks displayed increased body weight gain and liver and fat mass compared to WT mice (Fig. 2A–C), despite similar daily food intake (Fig. 2D). In line with what was observed with regular chow diet, fasting plasma TG and insulin levels as well as HOMA-IR were significantly higher in LFD-fed PHD1−/− mice compared to WT mice (Fig. 2E,H,I), indicating impairment in whole-body metabolic homeostasis. Furthermore, intraperitoneal GTT demonstrated increased glucose intolerance and higher plasma insulin levels in LFD-fed PHD−/− mice compared to their WT counterparts (Fig. 2J–L). Of note, the differences in body weight gain, plasma parameters and whole-body metabolic homeostasis in between genotypes were already observed after 6 weeks of synthetic diet (Fig. S2). Overall, these results demonstrate that PHD1−/− mice on LFD maintain an adverse metabolic phenotype compared to WT mice.

As expected, WT mice fed a HFD for 12 weeks exhibited increased body weight, liver and fat mass and fasting plasma TC, glucose and insulin levels (Fig. 2A–H). HOMA-IR and whole-body glucose tolerance were also significantly impaired compared to mice fed a LFD (Fig. 2I–L). Surprisingly, although HFD also promoted metabolic alterations in PHD1−/− mice, the effect of the diet on whole-body glucose homeostasis seemed somewhat dampened in those mice. Indeed, PHD1−/− mice fed a HFD displayed significantly lower fasting plasma glucose levels (Fig. 2G) and better glucose tolerance (Fig. 2J,K) than their WT counterparts.

### PHD1 deficiency impairs hepatic insulin sensitivity

**Figure 3:**
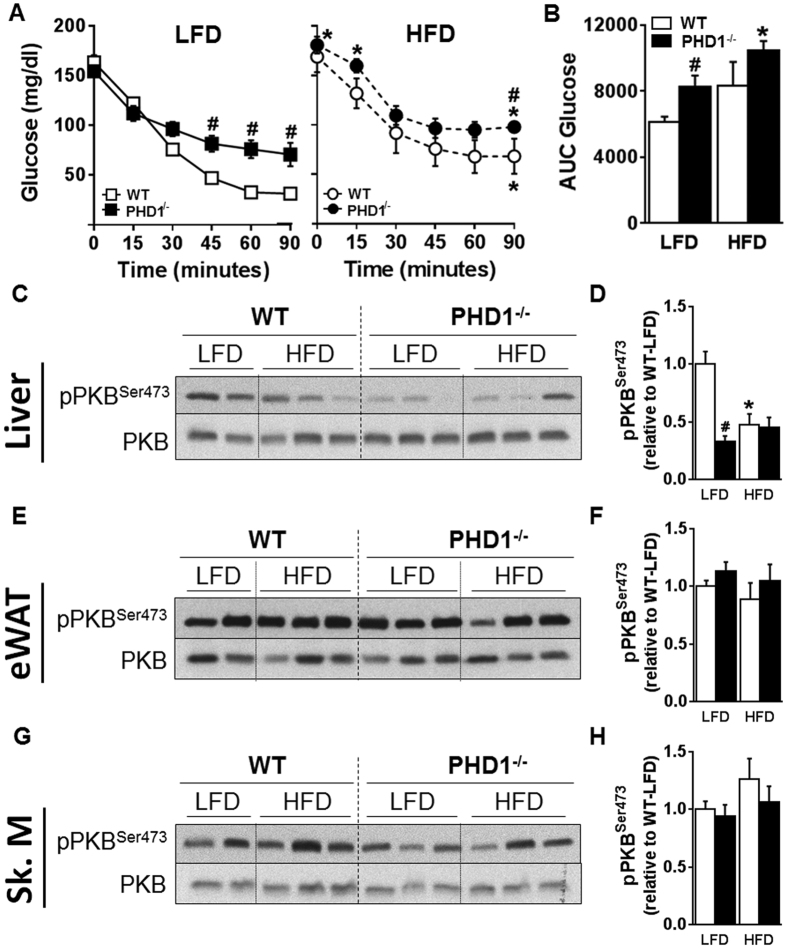
PHD1 deficiency induces systemic and liver-specific insulin resistance. An intraperitoneal ITT (0.5 U/kg total body weight) was performed in 6-hour unfed WT (open symbols/bars) and PHD−/− (black symbols/bars) mice after 11 weeks of either low-fat diet (LFD, squares) or high-fat (HFD, circles) diet. Blood glucose levels were measured at the indicated time-points (**A**) and the AUC of the glucose excursion curve was calculated as a measure of insulin resistance (**B**). In separate experiments, mice were sacrificed 15 min after insulin injection and tissue-specific insulin signaling was studied in liver, eWAT and skeletal muscle (Sk. M) by Western blot. Representative blots are shown in (**C,E,G**). Densitometric quantification was performed and results were expressed as fold change relative to WT-LFD mice (**D,F,H**). Data are means ± SEM (n = 4 for LFD-WT; n = 7 for LFD-PHD1−/−; n = 5 for HFD-WT; n = 7 for HFD-PHD1−/−). *p < 0.05 *vs* LFD mice, ^#^p < 0.05 *vs* WT mice.

In order to study the impact of PHD1 deletion on systemic insulin sensitivity, LFD- and HFD-fed WT and whole-body knockout mice were subjected to an intraperitoneal ITT. The hypoglycemic response to insulin was impaired in LFD-fed PHD1−/− mice compared to WT mice (Fig. 3A,B), reflecting systemic insulin resistance. In parallel experiments, metabolic tissues (liver, eWAT and skeletal muscle) were harvested 10 min after insulin administration to assess tissue-specific insulin sensitivity by Western blot. Remarkably, the insulin-induced phosphorylation of protein kinase B (PKB) was significantly reduced in the liver of PHD1−/− compared to WT mice on LFD (Fig. 3C,D) whereas no significant effects were observed in eWAT and skeletal muscle (Fig. 3E–H), indicating that the alteration of systemic insulin sensitivity in LFD-fed PHD1−/− mice was mostly due to hepatic insulin resistance. When subjected to HFD, both WT and PHD−/− mice developed systemic and hepatic insulin resistance but no significant differences were observed between the two genotypes (Fig. 3A–H). As the canonical insulin signaling pathway was not impaired in eWAT and skeletal muscle from PHD1 mice, we next investigated whether the AMP-activated protein kinase (AMPK) signaling, an insulin-independent pathway involved in the peripheral regulation of glucose homeostasis, was affected in these tissues. In skeletal muscle, AMPK activity, assessed by the pThr172-AMPKα/AMPKα ratio, was significantly higher in skeletal muscle from PHD1−/− mice on HFD than in those from WT mice whereas no differences were found in mice on LFD (Fig. S3A–D). In line with this, similar changes in the phosphorylation state of Acetyl-CoA Carboxylase (ACC), one of the main downstream targets of AMPK, were observed (Fig. S3A,E,F). Of note, protein expression of AMPKα and ACC was found to be increased and decreased, respectively, in skeletal muscle of PHD1−/− mice, whatever the nutritional conditions (Fig. S3A–G). In eWAT, AMPK activity was lower in both LFD- and HFD-fed PHD1−/− mice when compared to WT mice (Fig. S4A–D). As expected, protein expression of both ACC and Fatty Acid Synthase (FAS) was reduced by HFD in both genotypes but was found to be significantly lower in LFD-fed PHD1−/− mice when compared to WT mice (Fig. S4E,F). Of note, mRNA levels of key genes involved in adipose tissue biology were comparable in eWAT from WT and PHD1−/− mice (Fig. S4G), with the notable exception of *Lep* (Leptin) and *Ucp1* that were significantly higher in PHD1-deficient mice, in line with the larger adipose mass observed in those mice. Furthermore, PHD1 deficiency did not affect the expression of inflammatory genes in eWAT from LFD-fed mice (Fig. S4H).

### PHD1 deficiency promotes hepatic steatosis and liver inflammation

**Figure 4:**
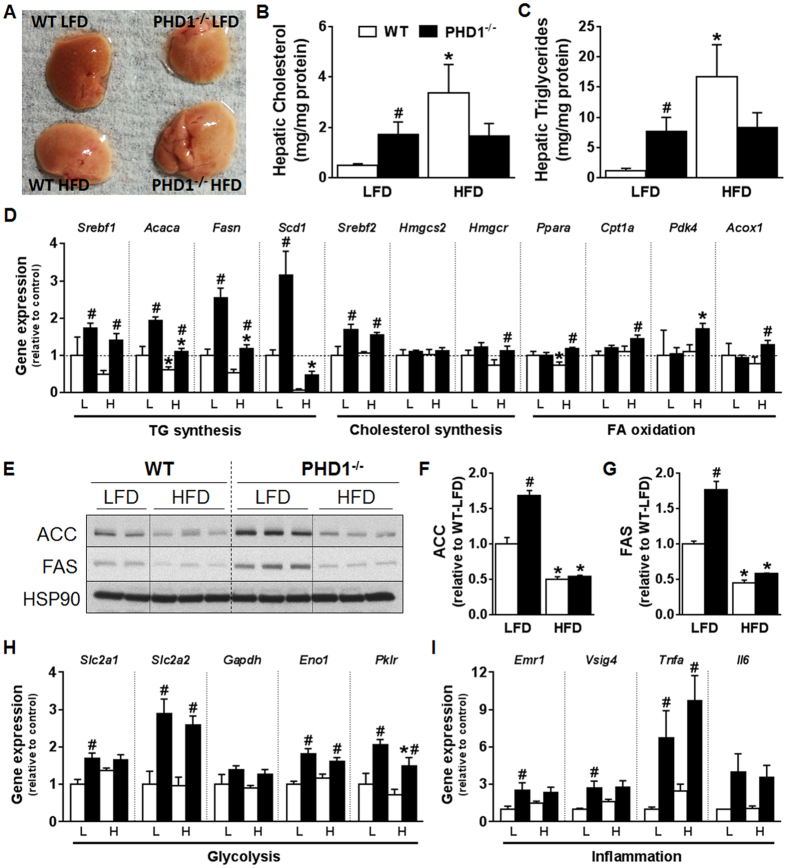
PHD1 deficiency promotes hepatic steatosis. Livers from WT (open bars) and PHD−/− (black bars) mice on either low-fat (LFD) or high-fat (HFD) diet were sampled (**A**) after 12 weeks. Hepatic cholesterol (**B**) and triglycerides (TG, **C**) contents were determined. The mRNA expression of key genes involved in the regulation of hepatic TG synthesis (*Srebf1*: SREBP-1c; *Acaca*: ACC1; *Fasn*: FAS; *Scd1*: SCD1), cholesterol synthesis (*Srebf2*: SREBP2 ; *Hmgcs2*: HMGCoA synthase; *Hmgcr*: HMGCoA reductase) and fatty acid oxidation (*Ppara*: PPARα; *Pdk4*: PDK4 ;*Cpt1a*: CPT-1α; *Acox1*: acyl-coA oxidase 1) was measured by RT-qPCR (**D**). Liver ACC and FAS protein expression were studied by Western blot. Representative blots are shown in (**E**). Total protein expression was quantified by densitometric analysis and expressed as fold change relative to WT-LFD mice (**F,G**). HSP90 was used for internal housekeeping protein expression. The mRNA expression of key genes involved in hepatic glycolysis (H; *Slc2a1:*GLUT1*; Slc2a2,* GLUT2*; Gapdh,* GAPDH*; Eno1,* Enolase*; Pklr,* PK) and inflammation (I; *Emr1:* F4/80; *Vsig4:* VSIG4; *Tnfa:* TNFα*; Il6:* IL6) was measured by RT-qPCR. All the RT-qPCR results are expressed relative to the housekeeping gene RPLP0 as fold change *vs* WT-LFD mice. Data are means ± SEM (n = 4 for LFD-WT; n = 7 for LFD-PHD1−/−; n = 5 for HFD-WT; n = 7 for HFD-PHD1−/−). *p < 0.05 *vs* LFD mice, ^#^p < 0.05 *vs* WT mice.

Hepatic insulin resistance is often associated with nonalcoholic steatohepatitis (NASH), which is characterized by inflammation and ectopic accumulation of TG in the liver. Strikingly, PDH1−/− mice on LFD exhibited visible steatosis and significantly higher hepatic cholesterol and TG content (Fig. 4A–C). This was associated with increased gene and protein expression of the lipogenic enzymes ACC and FAS (Fig. 4D–G) compared to WT mice. Of note, similar increase in lipogenic gene expression were also found in PDH1−/− mice on regular chow diet when compared with WT mice (Fig. S5). Interestingly, the expression of key genes involved in glycolysis were also upregulated in the liver from PDH1−/− mice (Fig. 4H). In addition, a higher expression of some inflammatory gene markers was observed in liver from LFD-fed PHD1−/− mice (Fig. 4I). As expected, HFD increased liver cholesterol and TG levels in WT mice, along with a compensatory down-regulation of proteins involved in hepatic *de novo* lipogenesis (Fig. 4A–I). However, neither hepatic lipid composition nor expression of lipogenic proteins significantly differed between WT and PHD1−/− mice on HFD, indicating that HFD-induced hepatic steatosis was not aggravated by PHD1 deficiency.

Overall, our results indicate that whole-body PHD1 deficiency in mice promotes adiposity, hepatic steatosis and liver-specific insulin resistance but does not worsen the deleterious effects of HFD on metabolic homeostasis.

## Discussion

In the present study, we report that whole-body PHD1 deletion in mice impairs systemic glucose homeostasis and insulin sensitivity in mice on standard chow or low-fat diet, a detrimental metabolic phenotype associated with increased hepatic steatosis and inflammation and liver-specific insulin resistance. However, PHD1 deficiency does not worsen the deleterious effects of HFD on whole-body insulin sensitivity and metabolic homeostasis.

In our conditions, the main metabolic tissue apparently involved in the alteration of systemic insulin resistance and glucose intolerance in PHD1−/− mice on LFD appears to be the liver, where a very significant decrease in insulin signaling was evidenced. At the mechanistic level, an increase in both glycolytic and lipogenic enzymes, hepatic lipid content, and inflammatory markers were found in LFD-fed PHD1−/− mice. Interestingly, both hypoxia and PHD-1 deletion were shown to activate the pro-inflammatory IKKβ/NFkβ canonical pathway in an *in vitro* model of cancer cells^22^, suggesting that a similar alteration might occur in metabolic tissues and underlie increased local inflammation and insulin resistance. On the other hand, the increase in hepatic steatosis in PHD1−/− mice probably results from increased *de novo* lipogenesis, a metabolic process converting carbohydrate-derived acetyl-CoA produced during glycolysis into TG under the control of key enzymes involved in glycolytic and lipogenic pathways^23^,^24^. Indeed, the robust increase in the expression of glycolytic genes and lipogenic enzymes ACC and FAS likely contributes to the enhanced hepatic TG content observed in the liver from chow- and LFD-fed PHD1−/− mice. Interestingly, liver-specific deletion of the three PHD isoforms was also previously reported to induce severe hepatic steatosis^20^,^25^,^26^, although deletion of PHD1 or PHD2 alone and of a combination of them (1 + 2 and 2 + 3) did not seem sufficient to promote a significant increase in liver TG content in their experimental conditions^20^,^25^,^26^. However, in contrast to our results, enhanced hepatic lipid accumulation in the triple PHD knockout mice was associated with a decrease in mRNA expression of the lipogenic genes *Srebf1* (SREBP-1C) and *Fas* (FAS) whereas expression of the glycolytic gene *Slc2a1* (GLUT1) was similarly increased^20^. These conflicting results suggest that various PHD-specific downstream targets and/or pathways might be involved in the development of fatty liver.

The canonical function of PHDs is to hydroxylate HIF-α subunits, leading to its ubiquitination by the pVhl and subsequent degradation by the proteasome. Importantly, sustained activation of hepatic HIFs, either by overexpression of HIF-α isoforms or inactivation of the pVhl, has been previously shown to induce hepatic steatosis and inflammation^27^,^28^,^29^,^30^. It is noteworthy that, although disruption of pVhl activates HIF and leads to upregulation of both HIF-1α and HIF-2α target genes^31^,^32^, the induction of hepatic steatosis and inflammation seems to be exclusively HIF-2α-dependent in this model^29^,^30^. Indeed, liver-specific inactivation of pVhl and pVhl/HIF-1α promotes increase in hepatic triglyceride content^29^,^30^ and pro-inflammatory Il6 and Il1b gene expression^29^ whereas pVhl/HIF-2α mutant mice are phenotypically normal^29^,^30^.

The liver expresses all three HIF-α family members and whether the metabolic dysfunctions observed in the whole-body PHD1−/− mice, notably hepatic steatosis, was conferred by stabilization of specific HIFs remains to be elucidated. It has been shown that functional redundancies exist among the three PHD isoforms in targeting HIF-α subunits^11^,^33^, suggesting that PHDs, alone or in combination, might differently regulate HIF-1α or HIF-2α in the liver. Both HIF-1α and HIF-2α levels were found to be more abundant in the nucleus from PHD1−/− livers, with HIF-2α being the most upregulated isoform^34^. Due to lack of material, we were not able to assess this aspect in our present study but the increase in HIF-1α-specific target genes, such as *Slc2a2* (GLUT2), *Ldha* (LDH), *Eno1* (Enolase), *Pklr* (L-PK) and *Egln3* (PDH3)^35^, suggests a HIF-1α-dependent metabolic reprogramming in the liver of PHD1−/− mice promoting glycolysis and pyruvate metabolism. Taken together, the tissue-specific respective contribution of the various HIF-α isoforms in the metabolic phenotype of the PHD1−/− whole-body knockout mice remains to be investigated more extensively.

Although the liver seems to be a central player in the metabolic disorders induced by whole-body PHD1−/− deletion, we cannot exclude that additional defects in key pathways controlling nutrient metabolism in peripheral tissues might also occur in other metabolic organs. For instance, the reduction in both ACC and FAS protein expression and AMPK activity in WAT from PHD1−/− mice might contribute to impaired tissue-specific fat storage and oxidation, respectively, indirectly promoting ectopic fat accumulation in the liver. Further studies will be necessary to dissect the respective tissue contribution to the metabolic phenotype observed in whole-body PHD1−/− knockout mice.

As expected, WT mice developed obesity, hepatic steatosis and systemic insulin resistance when fed a HFD, and the extent of these diet-induced metabolic dysfunctions was found to be similar in PHD1−/− mice despite higher baseline body weight and insulin-resistance on standard chow diet. Surprisingly, the glucose tolerance was found to be slightly but significantly less impaired in PHD1−/− than in WT mice at both 6 and 12 weeks of HFD. Of note, PHD1−/− mice were previously reported to have tolerance to hypoxia, partly through metabolic reprogramming leading to a shift from oxidative toward anaerobic/glycolytic metabolism in skeletal muscle and liver^21^,^34^. A HIF-1-mediated increase in pyruvate dehydrogenase kinase 4 (PDK4) expression, which inhibits the conversion of pyruvate to acetyl-CoA and its subsequent entry into the mitochondrial tricarboxylic cycle, was proposed as one of the mechanisms underlying this metabolic adaptation^21^,^36^. Interestingly, in line with these observations, we also observed an increase in the expression of various glycolytic genes and a significantly higher PDK4 mRNA level in the liver of PHD1−/− mice. Altogether, it is therefore tempting to suggest that the higher glucose tolerance of HFD-fed PHD1−/− mice, compared to WT mice, might result from increased glucose uptake and oxidation in the liver, one of the main metabolic tissue contributing to systemic glucose homeostasis.

At the systemic level, the altered metabolic phenotype of our PHD1−/− mice on standard chow or LFD resembles that observed in a mouse model of adipose-tissue specific HIF-1 activation. Indeed, overexpression of a constitutively active form of HIF-1α in WAT led to an increase in body weight gain, fat mass and impairment of whole-body glucose tolerance in mice on standard chow diet^37^. However, in contrast with our study, the metabolic phenotype of these mice was clearly worsened when challenged with HFD, an effect associated with higher inflammation, fibrosis and insulin resistance in WAT^37^. In our condition, we rather observed a tendency toward decreased HFD-induced WAT inflammation in PHD1−/− mice, as evidenced by downregulation of some pro-inflammatory macrophage gene markers like *CD68* and *ITGAX* (Fig. S4). Interestingly, a recent study reports that both systemic and adipose-tissue specific deletion of PHD2 can protect mice against HFD-induced metabolic disorders^18^. In addition, liver-specific deletion of PHD3 alone or concomitantly with PHD1 or 2, was also shown to improve glucose tolerance and insulin sensitivity^20^. Adipocyte-specific PHD2−/− mice on HFD are indeed characterized by a decrease in fat mass and adipocyte size when compared to WT mice, as well as by a reduction in macrophage infiltration, expression of pro-inflammatory cytokines and lipogenesis in WAT^18^,^19^. Moreover, an increase in glucose oxidation in WAT due to up-regulation of glycolytic genes in adipocytes might also contribute to the improvement in whole-body glucose homeostasis^18^,^19^. A reduction in both HFD-induced hepatic gluconeogenesis and liver steatosis was also suggested to be involved in the favorable metabolic profile of these various models of PHD deficiency^18^,^19^,^20^. Of note, we did not find any significant differences in hepatic gluconeogenic gene expression between the two genotypes, whatever the diet used (data not shown). Overall, such contrasting results might originate from the specific PHD deletion used in these different studies. We herein displayed the metabolic consequences of whole-body PHD1 deletion, which might differ from those induced by a liver-specific deletion. For example, it can be speculated that part of the detrimental effect of whole-body PHD1 deletion on metabolic homeostasis might be secondary to a central effect, *i.e.* alteration of the hypothalamic regulation of peripheral insulin sensitivity and nutrient metabolism. Some HIF-α isoforms are indeed expressed in various hypothalamic regions^38^ and PHD1 deletion might therefore affect HIF-α protein content and transcriptional activity in key brain area involved in metabolic homeostasis. Interestingly, PHD1 was recently identified as a regulator of neuronal metabolism by regulating glucose flux through glycolytic and pentose phosphate pathways^39^. In addition, insulin signaling in the brain was also reported to protect against ectopic lipid accumulation in the liver by stimulating hepatic TG secretion^40^, suggesting that alteration of central insulin sensitivity in PHD1−/− mice might also contribute to hepatic steatosis. Further studies are clearly required to investigate these brain-mediated aspects.

Altogether, the reason(s) behind the discrepancies in the metabolic phenotype displayed by the various PHDs deletion mice models are not clear but may be due to differences and/or redundancies in the tissue-specific control of the various HIF-α isoforms by each PHD. Of note, most of the studies showing an improvement in metabolic homeostasis have been performed in HFD-fed PHD knockout mice, a condition where we also observed a trend for a better glucose tolerance in our whole-body PHD1−/− mice compared to WT mice. However, in contrast with our study, there is relatively few information on the metabolic phenotype of these animals on LFD or standard chow diet. Finally, it is important to underline that in our model of constitutive whole-body knockout, we observed some tissue-specific changes in expression of the other PHD isoforms when PHD1 was deleted, notably in the liver. Therefore, we cannot exclude that some of the detrimental effects on metabolic homeostasis found in our model are actually secondary to a shift in the composition (and function) of PHD isoforms in the various metabolic tissues. In addition, it cannot be excluded that molecules other than HIFs, notably among those involved in the regulation of cellular glucose and lipid metabolism, can also be potential downstream targets of PHDs, for example by direct hydroxylase-independent interactions with their amino-terminal domains^35^,^36^.

In conclusion, we report here that whole-body PHD1 deficiency promotes hepatic steatosis and liver-specific insulin resistance but does not worsen the deleterious effects of HFD on metabolic homeostasis. Further studies using tissue-specific and inducible deletion of PHD1 are clearly required for investigating the respective contribution of the organs involved in the regulation of metabolic homeostasis, including the central nervous system, in the HIF-dependent or independent impairment of whole-body insulin sensitivity and glucose homeostasis.

## Methods

### Ethics

Animal experiments were performed in accordance with the Guide for the Care and Use of Laboratory Animals of the Institute for Laboratory Animal Research and have received approval from the ethical committee of Université Grenoble Alpes (agreement number: B3851610006).

### Animals and experimental design

PHD1 (*Egln2*)−/− mice (Swiss × sv129 background) were generated in Peter Carmeliet’s group (VIB, Leuven, Belgium), as previously described^21^, and breeding couples were kindly provided for colony expansion. In the present study, PHD1−/− mice and their wild-type (WT) littermates were housed under standard conditions in conventional cages with *ad libitum* food and water. Ambient temperature was maintained at 20–22 °C. In the first experiment, male mice were fed a standard chow diet (RM1, Special Diets Services, Essex, England) and studied at 10 to 12 weeks of age. In the second set of experiments, 10 week-old male PHD1−/− and WT mice were fed a low-fat (10% energy derived from fat; D13091501; Research Diets) or a high-fat (60% energy derived from fat; D13091502; Research Diets) diet during 12 weeks.

### Plasma analysis

Blood samples were obtained in 6-hour unfed mice (food withdrawn at 8:00 am) via tail vein bleeding. Blood glucose levels were determined using a glucometer (OneTouch ultra, Lifescan, Issy-Les-Moulineaux, France). Plasma TC, TG and insulin levels were measured using the commercially available enzymatic kits 236691, 11488872 (Roche Molecular Biochemicals, Indianapolis, IN) and ELISA insulin kit (#EZRMI, Milipore), respectively. The homeostatic model assessment (HOMA) adapted to mice was calculated as ([glucose (mg/dl)*0.055] × [insulin (ng/ml) × 172.1])/3857 and used as a surrogate measure of whole-body insulin sensitivity^41^.

### Glucose and insulin tolerance tests

All the experiments were performed in 6-hour unfed mice (food withdrawn at 8:00 am). Whole-body glucose tolerance and insulin sensitivity were assessed by intraperitoneal (i.p.) glucose tolerance (GTT) and insulin tolerance (ITT) tests. After an initial blood collection (t = 0), i.p. injections of glucose (2g/kg total body weight) or insulin (0.5U/kg total body weight, NovoRapid, Novo Nordisk, Bagsvaerd, Denmark) were performed in conscious mice. Blood glucose levels were next measured by tail bleeding at 15, 30, 60, 90 and 120 min using a glucometer. The glucose or insulin areas under the curve (AUC) were measured using trapezoidal integration.

### Hepatic lipid composition

Liver lipids were extracted as previously described^42^. Briefly, small liver samples were homogenized in ice-cold methanol. After centrifugation, lipids were extracted by addition of 1800 μl CH_3_OH:CHCl_3_ (1:3 v/v) to 45 μl homogenate, followed by vigorous vortexing and phase separation by centrifugation (14000 rpm; 15 min at RT). The organic phase was dried and dissolved in 2% Triton X-100 in water. TG and cholesterol concentrations were measured using commercial kits as described above. Liver lipids were expressed as mg per mg protein, which was determined using the Bradford protein assay kit (Sigma-Aldrich, Saint-Quentin Fallavier, France).

### Western blot analysis

Snap-frozen liver, skeletal muscle and epididymal WAT samples (~50 mg) were lysed in ice-cold buffer containing: 50 mM Hepes (pH 7.6), 50 mM NaF, 50 mM KCl, 5 mM NaPPi, 1 mM EDTA, 1 mM EGTA, 1 mM DTT, 5 mM β-glycerophosphate, 1 mM sodium vanadate, 1% NP40 and protease inhibitors cocktail (Complete, Roche, Mijdrecht, The Netherlands). Western blots were performed as previously described^42^. Primary antibodies used are listed in Supplementary Table 1. Bands were visualized by enhanced chemiluminescence and quantified using Image J (NIH, US).

### RNA purification and qRT-PCR

RNA was extracted from snap-frozen liver, skeletal muscle or epididymal adipose tissue samples (~25 mg) using Trireagent RNA isolation reagent (Sigma, Aldrich, Saint-Quentin Fallavier, France). Total RNA (0.5 μg) was reverse-transcripted and quantitative real-time PCR was then performed with SYBR Green Core Kit on a MyIQ thermal cycler (Bio-Rad). mRNA expression was normalized to RPLP0 mRNA content and expressed as fold change compared to control mice using the ∆∆CT method. Primers sequences are listed in Supplementary Table 2.

### Statistical analysis

All data are expressed as mean ± SEM. Statistical analysis was performed using Graphpad Prism 6 software package for Windows (San Diego, California USA) with two-tailed unpaired Student’s test (WT *vs* PHD1−/− on standard chow diet) or two-way ANOVA with multiple comparisons followed by post hoc Fisher’s LSD test (WT *vs* PHD1−/− on either LFD or HFD). Differences between groups were considered statistically significant when p < 0.05.

## Additional Information

**How to cite this article**: Thomas, A. *et al.* Hypoxia-inducible factor prolyl hydroxylase 1 (PHD1) deficiency promotes hepatic steatosis and liver-specific insulin resistance in mice. *Sci. Rep.* **6**, 24618; doi: 10.1038/srep24618 (2016).

## Supplementary Material

Supplementary Information
