# Supplementary material for: Hypoxia-inducible factor prolyl hydroxylase 1 (PHD1) deficiency promotes hepatic steatosis and liver-specific insulin resistance in mice
Source: Sci Rep. 2016 Apr 20;6:24618. doi: 10.1038/srep24618 (PMC4837354; doi:10.1038/srep24618)
Supplement: Supplementary Information [file srep24618-s1.pdf]

Hypoxia-inducible factor prolyl hydroxylase 1 (PHD1) deficiency promotes hepatic steatosis and liver-specific insulin resistance in mice

Amandine Thomas, Elise Belaidi, Judith Aron-Wisnewsky, Gerard C. van der Zon, Patrick Levy, Karine Clement, Jean-Louis Pepin, Diane Godin-Ribuot and Bruno Guigas

Supplementary Figure 1. Effects of whole-body PHD1 deficiency on tissue-specific expression of PHDs isoforms. The mRNA expression of the various HIF-PHDs isoforms (*Egln2*: PHD1; *Egln1*: PHD2; *Egln3*: PHD3; *Ldha*: LDHA) was measured by RT-qPCR in liver (A), epididymal white adipose tissue (B) and skeletal muscle (C) of WT (open bars) and PHD1$^{-/-}$ (black bars) mice on standard chow diet. The results are expressed relative to the housekeeping gene RPLP0 as fold change *vs* WT mice. Data are means ± SEM (n=7 for WT; n=13 for PHD1-/-). # p<0.05 *vs* WT mice.

Supplementary Figure 2. PHD1 deficiency promotes weight gain and insulin resistance but does not worsen high-fat diet-induced metabolic alterations. WT (open bars) and PHD1-/- (black bars) mice were fed a low-fat (LFD, 10 % fat) or high-fat (HFD, 45% fat) diet for 6 weeks. Body weight was monitored throughout the experimental period (A). Delta (Δ) change in body weight from the start of diet (B), plasma triglycerides (C), total cholesterol (D), glucose (E) and insulin (F) levels were determined and HOMA-IR (G) was calculated in 6-hour unfed mice at week 6. An intraperitoneal GTT (2 g/kg of total body weight) was performed in 6-hour unfed mice at week 5. Blood glucose levels were measured at the indicated time-points (H), and the area under the curve (AUC) of the glucose excursion curve was calculated as a measure of glucose tolerance (I). The plasma insulin level during ipGTT was measured at 15 minutes (J). An intraperitoneal ITT (0.5 U/kg total body weight) was performed in 6-hour unfed mice at week 6. Blood glucose levels were measured at the indicated time-points (K) and the AUC of the glucose excursion curve was calculated as a measure of insulin resistance (L). Data are means ± SEM (n=4 for LFD-WT; n=7 for LFD-PHD1-/-; n=5 for HFD-WT; n=7 for HFD-PHD1-/-). * p<0.05 *vs* LFD-fed mice, # p<0.05 vs WT mice.

Supplementary Figure 3. Effects of PHD1 deficiency on AMPK signaling pathway in skeletal muscle from low- and high-fat diet-fed mice. Protein expression of AMPKα and ACC, and phosphorylation state of Thr172-AMPKα and Ser79-ACC were assessed by Western blot 15min after insulin injection in skeletal muscle from WT (open bars) and PHD1-/- (black bars) mice on low-fat (LFD) or high-fat (HFD) diet, as described in Figure 3. Representative blots are shown in (A) and densitometric quantifications in (B-G). Phospho/total ratios were calculated and expressed as fold change relative to WT-LFD mice. Data are means ± SEM (n=4 for LFD-WT; n=7 for LFD-PHD1-/-; n=5 for HFD-WT; n=7 for HFD-PHD1-/-). * $p<0.05$ *vs* LFD mice, # $p<0.05$ *vs* WT mice

Supplementary Figure 4. Effects of PHD1 deficiency on AMPK signaling and expression of lipogenic proteins and metabolic/inflammatory genes in white adipose tissue from low- and high-fat diet-fed mice. Protein expression of AMPKα, ACC and FAS, and phosphorylation state of Thr172-AMPKα were assessed by Western blot 15min after insulin injection in epididymal white adipose tissue (eWAT) from WT (open bars) and PHD1-/- (black bars) mice on low-fat (LFD) or high-fat (HFD) diet, as described in Figure 3. Representative blots are shown in (A) and densitometric quantifications expressed as fold change relative to WT-LFD mice in (B-F). HSP90 was used for internal housekeeping protein expression. mRNA expression of key genes involved in glucose/FA uptake (*Insr*: Insulin receptor β, *Slc2a4*: GLUT4; *Cd36*: CD36), triglyceride synthesis (*Fasn*: FAS; *Acaca*: ACC1), fatty acid oxidation (*Lipe:* HSL*; Cpt1a*: CPT1α; *Ucp1*: UCP1) and adipokines (*Adipoq*: Adiponectin; *Lep*: Leptin) was measured by RT-qPCR (G). mRNA expression of key genes involved in eWAT inflammation (H) was measured by RT-qPCR (*Emr1:* F4/80,

*Cd68:*CD68*;Arg1:* ARG1*; Itgax:* CD11c*; Ccl2:* MCP-1; Il1b: IL1β*; Tnfa: TNFα).* Results are expressed relative to the housekeeping gene RPLP0 as fold change *vs* WT-LFD mice. Data are means ± SEM (n=4 for LFD-WT; n=7 for LFD-PHD1-/-; n=5 for HFD-WT; n=7 for HFD-PHD1-/-). * p<0.05 *vs* LFD mice, # p<0.05 *vs* WT mice.

Supplementary Figure 5. PHD1 deficiency increases hepatic lipogenic gene expression in chow-fed mice. Livers from WT (open bars) and PHD-/- (black bars) mice on standard chow diet. The mRNA expression of key genes involved in the regulation of hepatic TG synthesis (A; *Srebf1*: SREBP-1c; *Acaca*: ACC1; *Fasn*: FAS; *Scd1*: SCD1), cholesterol synthesis (A; *Srebf2*: SREBP2 ; *Hmgcr*: HMGCoA reductase; *Hmgcs2*: HMGCoA synthase) and fatty acid oxidation (A; *Ppara*: PPARα; *Pdk4*: PDK4 ;*Cpt1a*: CPT-1α; *Acox1*: acyl-coA oxidase 1) and glycolysis (B; *Gapdh*, GAPDH*; Eno1*, Enolase*; Pklr*, PK) was measured by RT-qPCR. The results are expressed relative to the housekeeping gene RPLP0 as fold change *vs* WT mice. Data are means ± SEM (n=7 for WT; n=13 for PHD1-/-). # p<0.05 *vs* WT mice.

Supplementary Figure 1

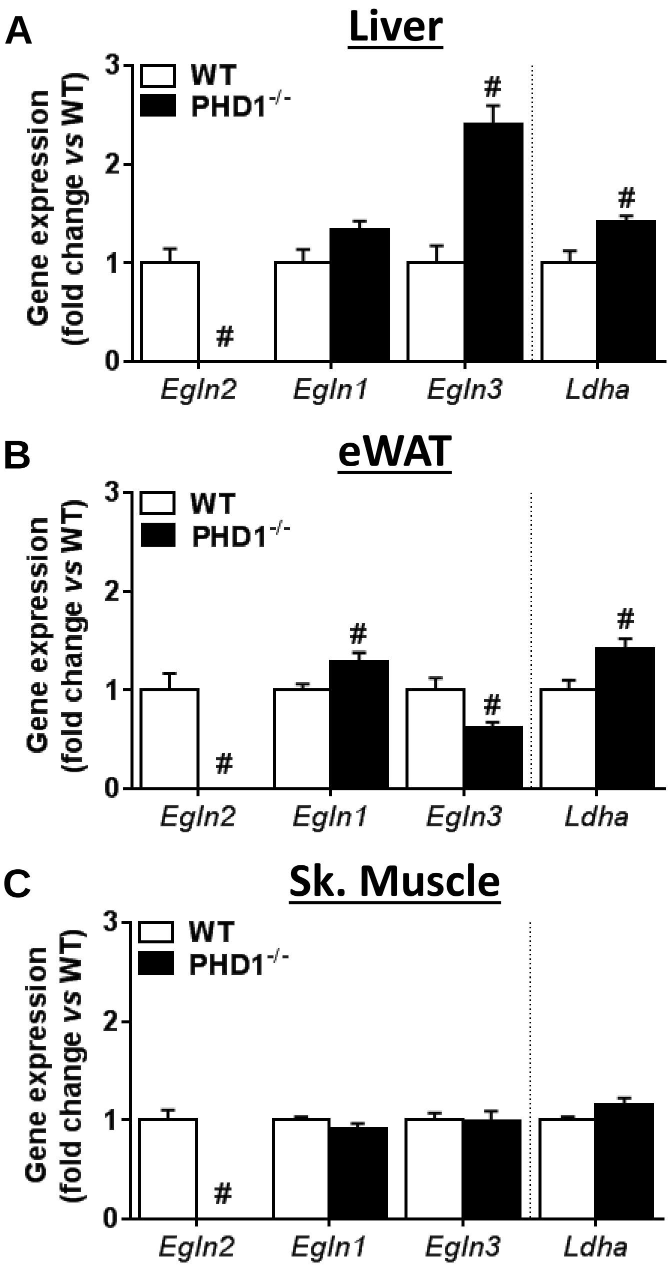

Supplementary Figure 2

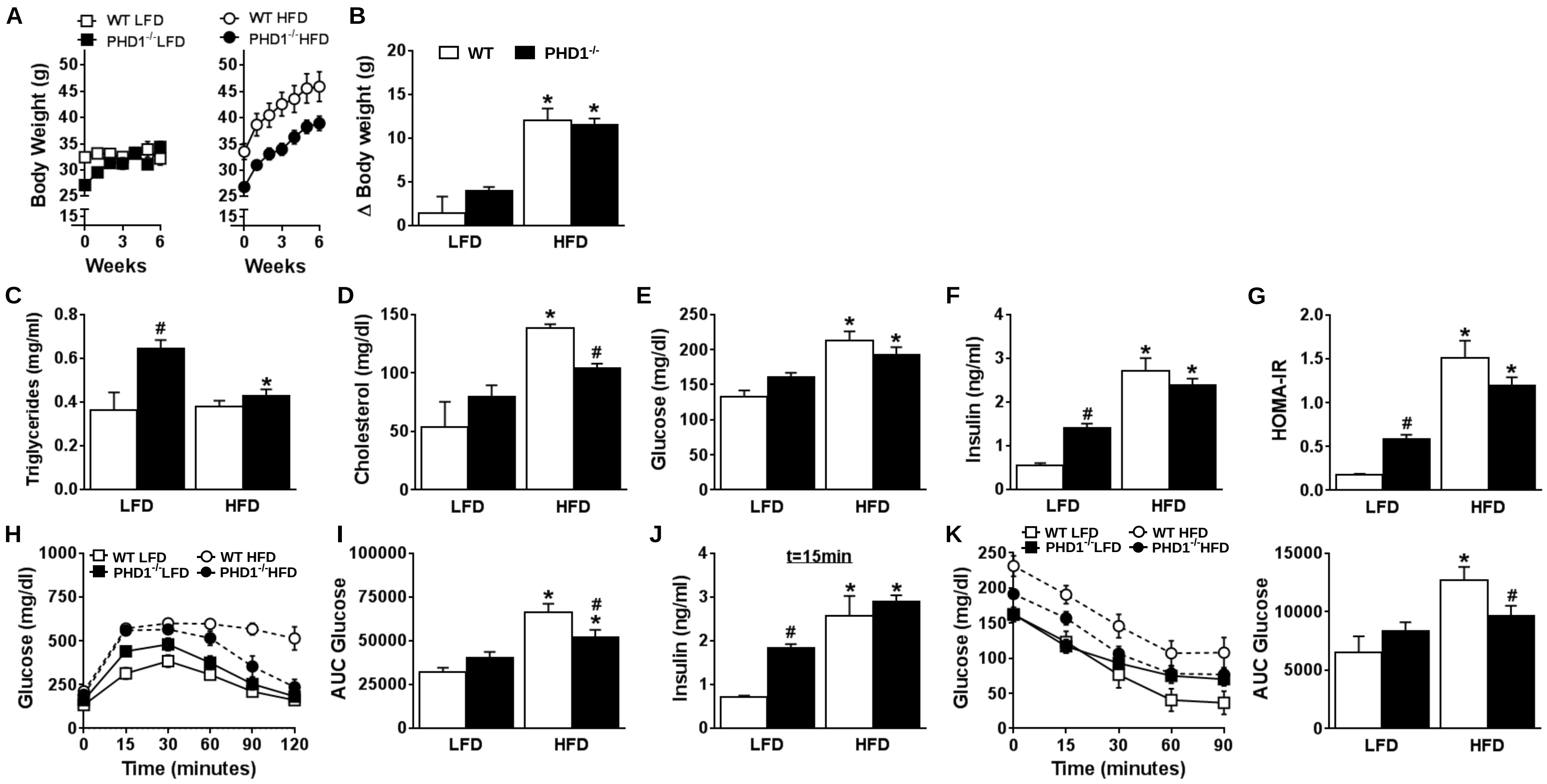

Supplementary Figure 3

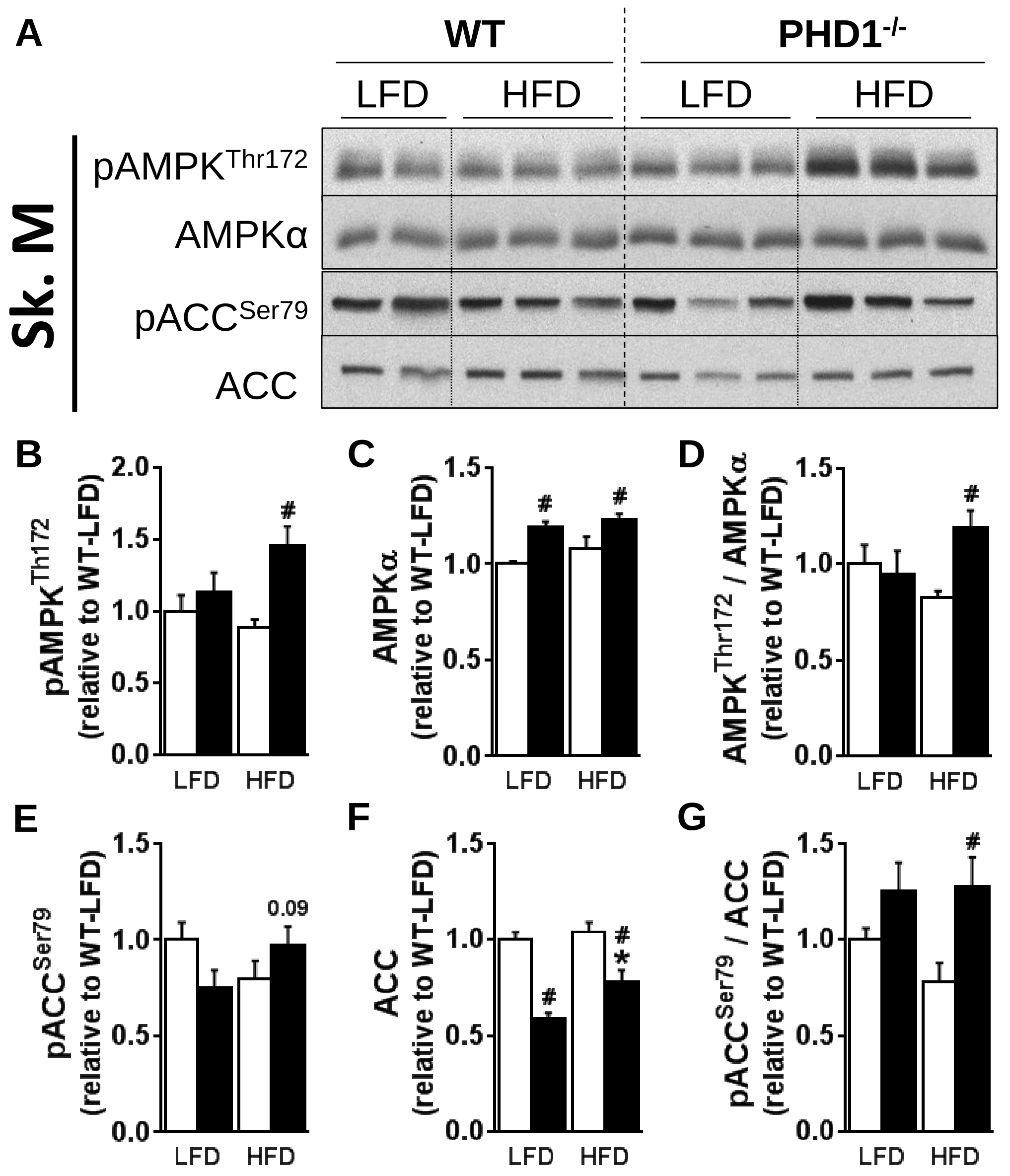

Supplementary Figure 4

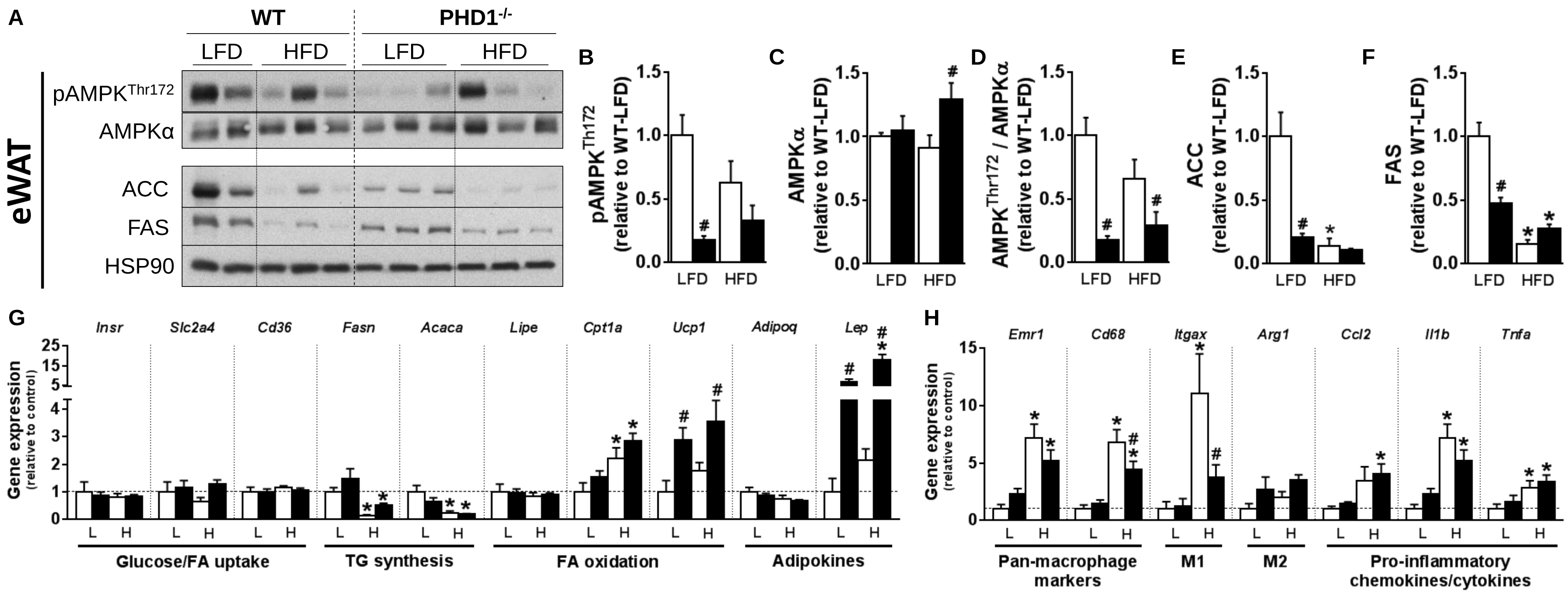

Supplementary Figure 5

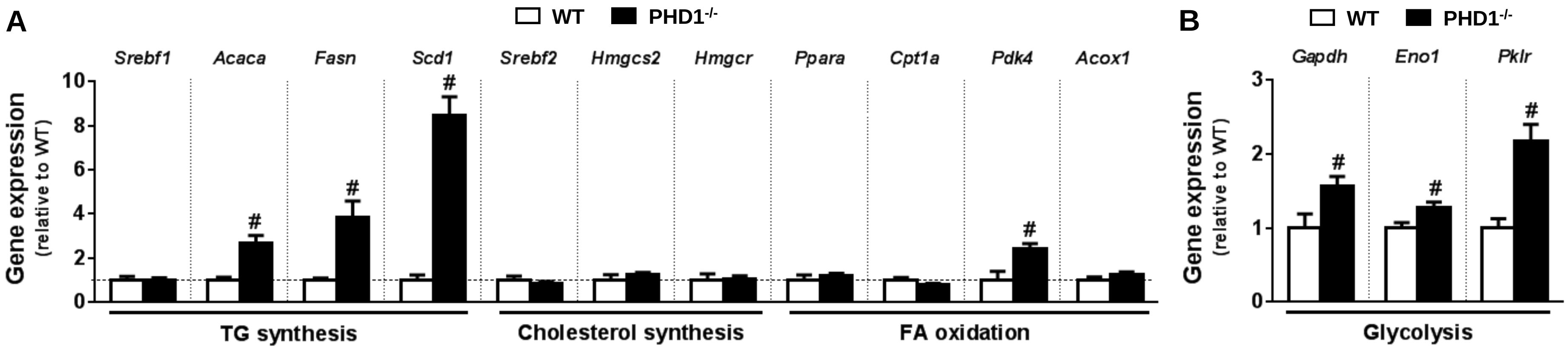

Supplementary Table 1: Primary antibodies for Western blots

Primary antibody	Residue	Supplier	Reference	Dilution
**ACC**	-	Cell Signaling	#3662	1:2000
**ACC**	Ser79	Cell Signaling	#3661	1:2000
**AMPKα**	-	Cell Signaling	#2532	1:1000
**AMPKα**	Thr172	Cell Signaling	#2535	1:1000
**FAS**		Cell signaling	#3180	1:1000
**PKBα+β**		Upstate	07-416 + 07-372	1:2000
**PKB**	Ser473	Cell signaling	#9271	1:1000
**HSP90**		Santa Cruz	sc-7947	1:1000

Supplementary Table 2: Primer sequences for qRT-PCR

Gene	Accession number	Forward primer	Reverse primer
Acaca	NM_133360.2	CAGCTGGTGCAGAGGTACCG	TCTACTCGCAGGTACTGCCG
Acox1	NM_015729	GGGACCCACAAGCCTCTGCCA	GTGCCGTCAGGCTTCACCTGG
Adipoq	NM_009605	GGAATGACAGGAGCTGAAGG	CGAATGGGTACATTGGGAAC
Arg1	NM_007482.3	GACCACGGGGACCTGGCCTT	ACTGCCAGACTGTGGTCTCCACC
Ccl2	NM_011333.3	TCAGCCAGATGCAGTTAACGCCC	GCTTCTTTGGGACACCTGCTGCT
Cd36	NM_001159558	GCAAAGAACAGCAGCAAAATC	CAGTGAAGGCTCAAAGATGG
Cd68	NM_009853.1	CCTCCACCCTCGCCTAGTC	TTGGGTATAGGATTCGGATTTGA
Cpt1a	NM_013495	AGGAGACAAGAACCCCAACA	AAGGAATGCAGGTCCACATC
Egln1	NM_053207	AGGCTATGTCCGTCACGTTG	TACCTCCACTTACCTTGGCG
Egln2	NM_053208	TCACGTGGACGCAGTAATCC	CGCCATGCACCTTAACATCC
Egln3	NM_028133	AGGCAATGGTGGCTTGCTAT	GACCCCTCCGTGTAACTTGG
Emr1	NM_010130.4	CTTTGGCTATGGGCTTCCAGTC	GCAAGGAGGACAGAGTTTATCGTG
Eno1	NM_023119.2	TGGAGAACAAAGAAGCACTGG	TGCCAGACCTGTAGAACTCG
Fasn	NM_007988	CACAGGCATCAATGTCAACC	TTTGGGAAGTCCTCAGCAAC
Gapdh	NM_008084.2	TGTGTCCGTCGTGGATCTGA	CCTGCTTCACCACCTTCTTGAT
Hmgcr	NM_008255	CTTGTGGAATGCCTTGTGATTG	AGCCGAAGCAGCACATGAT
Hmgcs2	NM_008256.4	CATCGCAGGAAGTATGCCCG	GCTGTTTGGGTAGCAGCTCG
Il1b	NM_008361	GACCCCAAAAGATGAAGGGCT	ATGTGCTGCTGCGAGATTTG
Il6	NM_031168.1	TGTGCAATGGCAATTCTGAT	CTCTGAAGGACTCTGGCTTTG
Insr	NM_010568.2	GCCAAAATTATCATTGGACCCC	CATCCGGCTGCCTCTTTCT
Itgax	NM_021334.2	GCCACCAACCCTTCCTGGCTG	TTGGACACTCCTGCTGTGCAGTTG
Ldha	NM_010699.2	CCTGTGTGGAGTGGTGTGAA	ATCACCTCGTAGGCACTGTC
Lipe	NM_010719	AGCCTCATGGACCCTCTTCT	GCCTAGTGCCTTCTGGTCTG
Lep	NM_008493	CCCTGTGTCGGTTCCTGTGGC	GCGGATACCGACTGCGTGTGT
Pdk4	NM_013743	GATTGACATCCTGCCTGACC	CAGGGCTTTCTGGTCTTCTG
Ppara	NM_011144	CAACCCGCCTTTTGTCATAC	CCTCTGCCTCTTTGTCTTCG
Pklr	NM_013631	CCTCTGCCTTCTGGATATCGAC	CGATGGTGGCAATGATGCT
Scd1	NM_009127.4	GCTCTACACCTGCCTCTTCGGGAT	TCCAGAGGCGATGAGCCCCG
Slc2a1	NM_011400.3	AGCATCTTCGAGAAGGCAGG	ACAACAAACAGCGACACCAC
Slc2a2	NM_031197.2	TCATGTCGGTGGGACTTGTG	CCCAAGGAAGTCCGCAATGT
Slc2a4	NM_009204	CTCAATGGTTGGGAAGGAAA	GAGGAACCGTCCAAGAATGA
Srebf1	NM_011480	CTGGCTGAGGCGGGATGA	TACGGGCCACAAGAAGTAGA
Srebf2	NM_033218.1	GCGTTCTGGAGACCATGGA	ACAAAGTTGCTCTGAAAACAAATCA
Tnfa	NM_013693	GTCCCCAAAGGGATGAGAAG	CACTTGGTGGTTTGCTACGA
Ucp1	NM_009463	TCAGGATTGGCCTCTACGAC	TGCATTCTGACCTTCACGAC
